# Retrospective genomic analysis of the first Lumpy skin disease virus outbreak in China (2019)

**DOI:** 10.3389/fvets.2022.1073648

**Published:** 2023-01-12

**Authors:** Yu-Rong Wei, Wen-Ge Ma, Ping Wang, Wen Wang, Xiao-Hui Su, Xue-Yun Yang, Xiao-Yun Mi, Jian-Yong Wu, Jiong Huang

**Affiliations:** ^1^Xinjiang Key Laboratory of Animal Infectious Diseases, Institute of Veterinary Medicine, Xinjiang Academy of Animal Science, Urumqi, China; ^2^Center for Animal Disease Prevention and Control of Xinjiang, Urumqi, China

**Keywords:** Lumpy skin disease virus, Illumina sequencing, phylogenetic analysis, genome sequence, genome alignment

## Abstract

Lumpy skin disease caused by Lumpy skin disease virus (LSDV) is a severe systemic disease affecting cattle and other ruminants. Lumpy skin disease was first reported in northwest China in August 2019 and has severely threatened the cattle breeding industry in China. However, there have been limited genomic studies of LSDV from the first outbreak and its subsequent epidemics. This study aims to characterize the comparative genomic evolution of the LSDV strain from the first outbreak in China. The etiological agent was isolated in a Madin-Darby bovine kidney cell culture and subsequently identified by PCR and Sanger sequencing of six selected genes. The genome sequence was determined using Illumina sequencing and analyzed through genome alignment and phylogenetic tree. The results showed that all six genes were successfully amplified and genetically clustered into LSDV. The virus presented the highest homology to strain China/GD01/2020, which shared 100% identities among 150 open reading frames (ORFs), and 97.1–99.7% identities among additional 6 ORFs. Bayesian inference tree analysis revealed that the virus shared a common ancestor with LSDV strains from China and Vietnam. The study provides an additional genomic data for LSDV tracking and control in China and neighboring countries.

## 1. Introduction

Lumpy skin disease (LSD) is a significant transboundary viral disease that affects cattle water buffalos and giraffe, and other ruminants (1–4). The disease is caused by the Lumpy skin disease virus (LSDV), a member of the family Poxviridae, genus Capripoxvirus (5, 6). The virus appears to be mechanically transmitted by blood-sucking arthropods such as flies, mosquitoes and ticks, and to a lesser extent by direct contact between cattle (7, 8). In addition, LSDV-contaminated milk, blood, nasal secretions, and saliva are alternative sources of infection through feeding or drinking routes (9). The affected animals mainly manifest fevers and nodular lesions, and they produce dramatically less milk and undergo weight loss (10). LSDV can cause a high incidence of 5–45% when introduced into a herd, and the case fatality rate ranges from 0.5 to 7.0% (11, 12). As a result, LSD poses a significant economic threat to the cattle-breeding industry.

LSD was initially described in Zambia in 1929 and identified as a communicable disease in the 1940's (6). In the 1950's, LSDV spread rapidly through Central and Eastern Africa. It then spread out of Africa into the Middle East in the 1980's (13). Since 2015, LSD outbreaks have occurred in countries neighboring China, such as Kazakhstan, and Russia (14, 15). In August 2019, the first outbreak of LSD was reported in the Xinjiang Uygur Autonomous Region, Northwest China, in which borders Kazakhstan (16). The disease was then reported in eight provincial administrative regions (Anhui, Fujian, Hong Kong, Guangdong, Jiangxi, Sichuan, Taiwan, and Zhejiang), resulting in 10 LSDV outbreaks in China (16–18). From January 2013 to July 2021, there were 28,442 LSDV outbreaks worldwide, resulting in 326,300 cases and 15,500 deaths (19). The global dissemination of LSDV has resulted in serious risk of this contagious disease affecting the large cattle population (more than 95.6 million cattle in stock) in China.

The genome of LSDV is a linear double-stranded 145–152 kb DNA molecule that contains 150–156 predicted open reading frames (ORFs). The first complete genome sequence was determined in 2001 from primary lamb testicle cells of the Neethling type strain 2,490, which contained 156 annotated genes (20). Of these encoded genes, the G protein-coupled chemokine receptor (GPRC) and the RNA polymerase 30 kDa subunit (RP030) were recognized as markers for differentiating the poxviruses at the family and genus levels (21, 22). In addition, the LSDV contains 90 core genes conserved in all chordopoxviruses, and they have been used for phylogenetic analyses (17). Since 2001, approximately 40 LSDV genome sequences of different origins have been sequenced and deposited in GenBank, but comparative genomic data and evolutionary studies are still limited.

The genomes of Chinese strains from Guangdong and Hong Kong in South China have been sequenced (17, 23), and previous studies of these circulating Chinese LSDV strains showed close genetic relationship with the LSDV/Russia/Saratov/2017 (accession no. MH646674.1) strain or Neethling vaccine strain based on single or multiple genes (11). Thus, the genetic relationships between the LSDV isolate present in the first outbreak in China and those of subsequent epidemics in China and other countries still needs to be clarified. In this study, we employed next-generation sequencing to obtain the complete genome sequence of the LSDV/China/XJ01/2019 strain isolated from the only cow that died during the first LSDV outbreak in China. We also performed a detailed genomic comparison of LSDV/China/XJ01/2019 and related genomic sequences reported before and after this outbreak. This study provides insight into the spread of LSDV during the epidemic in China.

## 2. Materials and methods

### 2.1. Specimens

In August 2019, the first ever LSD outbreak occurred in Ili Kazakh Autonomous Prefecture, Xinjiang Uygur Autonomous Region, Northwest China, which shares an approximately 50-km border with Kazakhstan (Supplementary Figure S1). During the outbreak, a Holstein cow was found to have died from LSD. After an examination and dissection of the dead dairy cow, a skin nodule sample was collected. It was then transported and shipped to the laboratory under cold conditions and immediately stored at −80°C for further testing. The owner of the animal was informed about the purpose and process of this study. The farmer agreed to allow the skin nodule samples to be collected from his died cow.

### 2.2. DNA extraction and amplification

The virus was cultured in Madin-Darby bovine kidney cell, and after three generations of culturing, the viral genome was extracted by using a QIAamp DNA Mini Kit (QIAGEN). The RPO30 and GPRC genes were amplified using the primers listed in Supplementary Table S1. Four additional primers (containing deletion or insertion sequences compared with Goatpox and Sheeppox viruses) were designed to amplify Ankyrin repeat protein (LSDV152), Interleukin-1 receptor-like protein (LSDV013), Putative alpha amanitin-sensitive protein (LSDV009) and Putative late transcription factor (LSDV076) genes that can be used to differentiate the genus Capripoxvirus referred to as the strain, LSDV/Russia/Saratov/2017 (accession no. MH646674.1) (Supplementary Table S1). These six genes were purified using Quick Gel columns (QIAGEN), and then ligated into pMD19-T (Tiangen Biotech) and transformed into Escherichia coli DH5α competent cells (Tiangen Biotech). Quintuplicate positive clones were extracted for Sanger sequencing (Sangon Biotech). Nucleotide sequences of the above six genes were downloaded from the National Center for Biotechnology Information, USA (Supplementary Table S2). Phylogenetic relatedness analyses were carried out using the MEGA 11 (https://megasoftware.net/) using the Maximum Likelihood method and the best fitting DNA model with 1,000 bootstrap replicates (24).

### 2.3. Genome sequencing and analysis

The LSDV genomic DNA was used for library construction and next-generation sequencing (Novagene). The raw reads were processed using a standard in-house pipeline (Novagene) to remove adapters, host sequences, chimeras, short reads, and low-quality reads. The clean data were further mapped to LSDV/Russia/Saratov/2017 (accession no. MH646674.1) by using Geneious prime software (25). The resulting contigs were assembled into a whole-genome sequence, which was then mapped onto a reference genome, resulting in a draft genome sequence. The clean reads were mapped to the gaps between the draft genome sequence and LSDV/Russia/Saratov/2017 (accession no. MH646674.1) using the medium sensitivity/Fast mode and iterated up to five times using Geneious prime 2020.0.3 software (25). The mapped sequences were used to generate consensus sequences to obtain the primary genomic sequence, which was then manually checked. Genome annotation were performed using GATU software (26) with the 20L81_Bang-Thanh VNM 20 (accession no. MZ577076.1) and Kubash KAZ 16 (accession no. MN642592.1) genomes as reference. The annotations were manually verified and curated using the Ugene software package (27).

The genome sequence of the strain LSDV/China/XJ01/2019 was aligned to a set of reference LSDV sequences retrieved from GenBank using ClustalX 2.1 (http://www.clustal.org/clustal2/). A phylogenetic tree was generated using the alignment and Bayesian approaches in MrBayes v. 3.2.7 (28) to evaluate the relationships between LSDV/China/XJ01/2019 and reference genome sequences in GenBank (Supplementary Table S3). Phylogenetic reconstruction was performed using the GTR evolutionary model including a Γ distribution and two runs of four chains each. The chain convergence was evaluated after 200,000 generations. Results were considered stable if an EES value was >200.

## 3. Results

### 3.1. Virus identification

**Figure 1:**
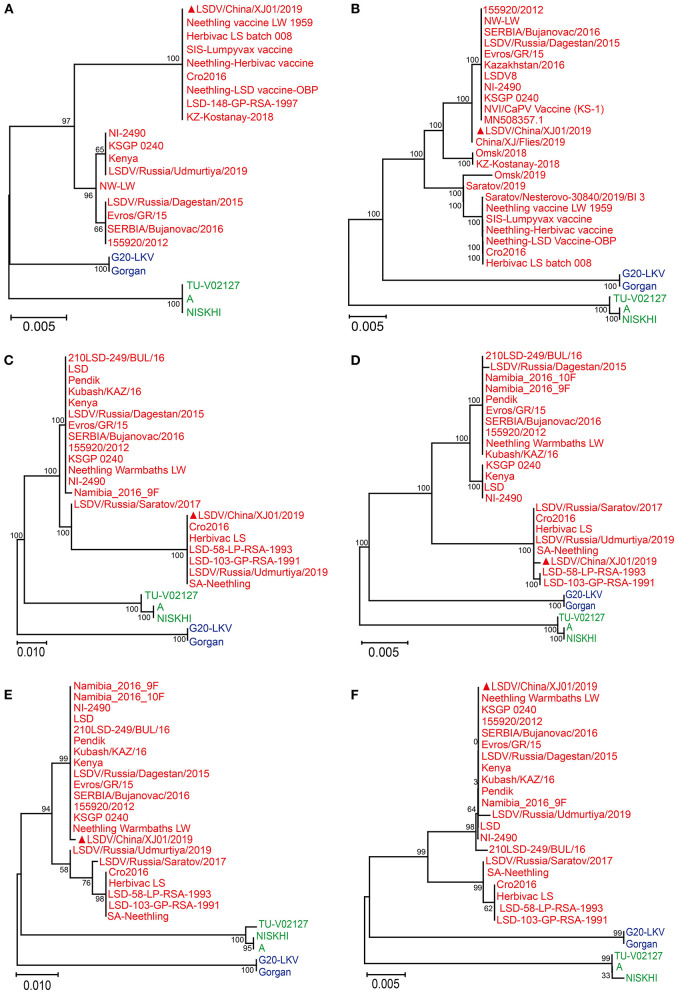
Phylogenetic diagram of RPO30 **(A)**, GPCR **(B)**, Putative alpha amanitin-sensitive protein **(C)**, Ankyrin repeat protein gene **(D)**, Interleukin-1 receptor-like protein gene **(E)** and Putative late transcription factor **(F)** genes of Lumpy skin disease virus. The phylogenetic tree was constructed using MEGA 11 with the Maximum Likelihood method. The Lumpy skin disease virus, Sheeppox virus, Goatpox virus are labeled with red, blue and green colors, respectively. The sequences obtained from this study are indicated by solid red triangles.

The GPCR and PRO30 genes were successfully amplified from the cell culture, primers for four other genes (Ankyrin repeat protein, Interleukin-1 receptor-like protein, Putative alpha amanitin-sensitive protein, and Putative late transcription factor) were designed to confirm the presence of LSDV, and finally, all six genes were tested as positive. After Sanger sequencing and phylogenetic analysis, all six genes were clustered with LSDV; consequently, the virus was identified as LSDV (Figure 1) and named LSDV/China/XJ01/2019.

### 3.2. Genome assembly

The complete genome sequence of the LSDV/China/XJ01/2019 strain was analyzed using an Illumina NovaSeq sequencer (Illumina, USA) generating 150-bp single reads. A total of 7,496,974 (150 × 150 bp) PE150 clean reads were obtained from the Illumina NovaSeq sequencer. Consensus sequences were generated by *de novo* assembling and mapping to LSDV/Russia/Saratov/2017 (accession no. MH646674.1). The clean reads were subsequently mapped to the consensus sequences and the average coverage was determined to be 728.6 × (Supplementary Figure S1). The viral genome sequence was then submitted and deposited in GenBank under accession no. OM105589.

### 3.3. Genomic comparisons between LSDV/China/XJ01/2019 and other strains in and around China

**Figure 2:**
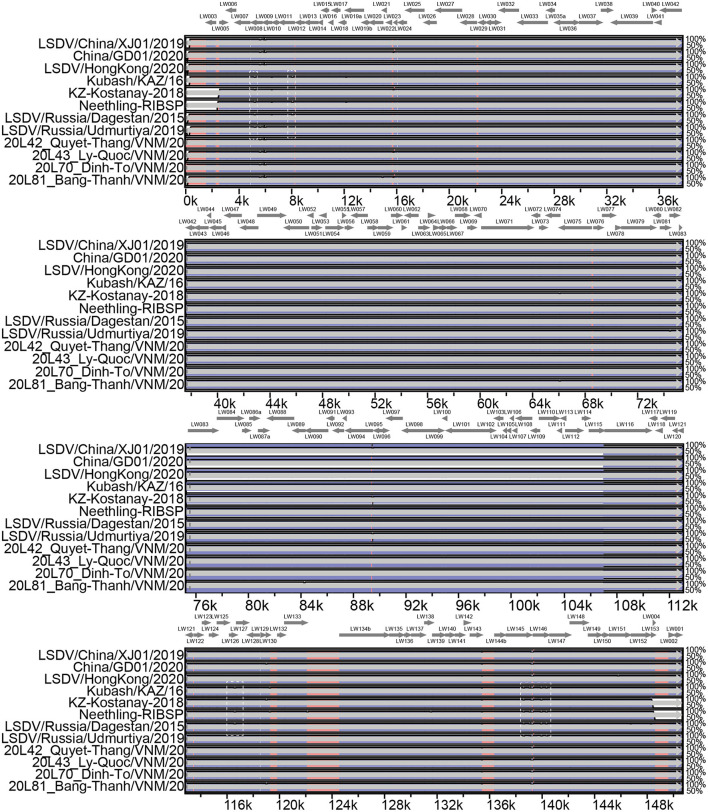
Multiple genomic sequence alignment of the LSDV strains in and around China. Graph showing sequence similarities between LSDV/China/XJ01/2019 and the strains 20L42_Quyet-Thang/VNM/20, 20L43_Ly-Quoc/VNM/20, 20L70_Dinh-To/VNM/20, 20L81_Bang-Thanh/VNM/20, China/GD01/2020, Kubash/KAZ/16, LSDV/China/XJ01/2019, LSDV/HongKong/2020, LSDV/Russia/Dagestan/2015, LSDV/Russia/Saratov/2017 and LSDV/ Russia/Udmurtiya/2019, which are plotted in a sliding 100-bp window. The seven differentiated regions between the genomes of the Chinese strains and reference strain LSDV/Russia/Saratov/2017 are indicated by white dotted boxes.

Pairwise genome sequence comparison revealed that LSDV/China/XJ01/2019 strain shared the highest similarity (99.9960%) with 20L43_Ly-Quoc/VNM/20, followed by 20L43_Ly-Quoc/VNM/20 (99.9954%), 20L42_Quyet-Thang/VNM/20 (99.9947%), 20L81_Bang-Thanh/VNM/20 (99.9947%) and China/GD01/2020 (99.9893%). The mVista program (http://genome.lbl.gov/vista/mvista/submit.shtml) was used to analyze the genome-wide differences among the strains in and around China. Seven regions of the viral genome were extremely variable, and it contained deletions and mutation mainly in genes LSDV008 (Putative soluble interferon-gamma receptor gene), LSDV011 (G protein-coupled chemokine receptor-like protein gene), LSDV126 (putative EEV glycoprotein gene), LSDV145 (Ankyrin repeat protein gene) and LSDV146 (Phospholipase D-like protein gene). Specifically, complete genome sequences of Chinese strains LSDV/China/XJ01/2019, China/GD01/2020, and LSDV/HongKong/2020 are greatly similar to the genomes of the Vietnamese strains 20L42_Quyet-Thang/VNM/20, 20L43_Ly-Quoc/VNM/20, 20L70_Dinh-To/VNM/20 and 20L81_Bang-Thanh/VNM/20, with only three significantly different regions observed (Figure 2).

### 3.4. Open reading frame comparisons of the LSDV/China/XJ01/2019 with other strains in and around China

The genome sequence was annotated referred to the strain 20L81_Bang-Thanh VNM 20 (accession no. MZ577076.1), a total of 156 open reading frames (ORFs) were identified in the strain, LSDV/China/XJ01/2019. Comparing the ORFs of LSDV/China/XJ01/2019 with the most closely related strain China/GD01/2020 revealed 150 ORFs sharing 100% sequence identities and 6 ORFs sharing 97.1–99.7% sequence identities (Table 1).

**Table 1:** ORFs that are not identical between LSDV/China/XJ01/2019 and other Chinese, Kazakhstan, Russian and Vietnamese Lumpy skin disease virus strains.

**Gene**	**Product**	**China**	**Vietnam**	**Kazakhstan**	**Russia**
LSDV001	Hypothetical protein	99.4	100	100	100
LSDV005	Interleukin-10-like protein	100	100	100	98.8–100
LSDV006	Interleukin-1 receptor-like protein	100	100	99.6–100	97.8–00
LSDV008	Putative soluble interferon gamma receptor	100	100	95.6–96.4	95.6–100
LSDV009	Putative alpha amanitin-sensitive protein	100	100	97.0	97.0–100
LSDV010	LAP|PHD-finger protein	100	100	98.8	98.8–100
LSDV011	G protein-coupled chemokine receptor-like protein	100	100	98.7–99.7	98.7–100
LSDV012	Ankyrin repeat protein	100	100	99.5–100	99.5–100
LSDV013	Interleukin-1 receptor-like protein	100	100	98.8–100	98.8–100
LSDV017	anti-apoptotic membrane protein	100	100	97.2–98.3	97.2–100
LSDV018	dUTPase	100	100	99.3	99.3–100
LSDV019	Kelch-like protein	100	100	99.3–100	99.3–100
LSDV020	Ribonucleotide reductase small subunit	100	100	99.7–100	99.7–10
LSDV021	Hypothetical protein	100	100	96.5–100	96.5–100
LSDV022	Hypothetical protein	100	100	97.4–100	97.4–100
LSDV024	S-S bond formation pathway protein	100	100	99.5–100	99.5–100
LSDV025	Ser-Thr kinase	99.8–100	100	100	99.8–100
LSDV026	Hypothetical protein	100	100	75.2–100	100
LSDV027	EEV maturation protein	100	100	99.5–100	100
LSDV028	Palmytilated EEV membrane glycoprotein	100	100	99.7–100	100
LSDV032	Poly(A) polymerase large subunit	100	100	99.6–100	99.6–100
LSDV033	Hypothetical protein	100	100	99.6–99.7	99.5–100
LSDV034	Double-strand RNA-binding protein	100	100	99.4–99.6	99.4–100
LSDV035	RNA polymerase subunit	100	100	99.4–100	100
LSDV036	Hypothetical protein	100	100	92.8–100	92.5–100
LSDV037	Hypothetical protein	100	100	92.8–99.8	99.8–100
LSDV038	Putative membrane protein	100	100	99.8–100	100
LSDV039	DNA polymerase	100	100	99.9–100	99.8–100
LSDV040	Sulfhydryl oxidase	100	100	99.9–100	100
LSDV041	Putative virion core protein	100	100	99.2–100	99.2–100
LSDV042	Hypothetical protein	100	100	99.2–99.6	99.4–100
LSDV043	Putative DNA-binding virion core protein	100	100	99.4–100	99.7–100
LSDV044	Hypothetical protein	100	100	99.7–100	100
LSDV045	Putative DNA-binding phosphoprotein	100	100	99.3–100	99.3–100
LSDV046	Putative IMV membrane protein	100	100	99.3–100	100
LSDV047	Hypothetical protein	100	100	99.5–100	99.7–100
LSDV048	Putative virion core protein	100	100	99.7–100	100
LSDV049	RNA helicase NPH-II	100	100	99.7–100	99.7–100
LSDV050	Putative metalloprotease	100	100	99.7–100	99.8–100
LSDV051	Hypothetical protein	100	100	99.1–100	99.1–100
LSDV052	Putative transcriptional elongation factor	100	100	99.1–100	100
LSDV054	Hypothetical protein	100	100	99.8–100	100
LSDV057	Putative virion core protein	100	100	99.7–100	99.7–100
LSDV058	Putative late transcription factor	100	100	99.7–100	100
LSDV059	Poxvirus myristoylprotein	99.7	99.7	99.7–100	99.7
LSDV060	Putative myristylated IMV envelope protein	100	100	99.7–100	100
LSDV061	Hypothetical protein	100	100	98.9–100	97.8–100
LSDV062	Hypothetical protein	100	100	98.9–100	100
LSDV064	Putative membrane protein	100	100	98.5–100	98.5–100
LSDV065	Hypothetical protein	100	100	98.5–100	98.6–100
LSDV066	Thymidine kinase	100	100	98.6–100	100
LSDV067	Putative host range protein	87.8–100	100	99.0–100	87.8–100
LSDV068	Poly(A) polymerase small subunit	100	100	99.0–100	99.7–100
LSDV069	RNA polymerase subunit	100	100	99.7–100	100
LSDV071	RNA polymerase subunit	100	100	99.9–100	99.9–100
LSDV072	Putative protein-tyrosine phosphatase	100	100	99.9–100	100
LSDV073	Putative viral membrane protein	100	99.5–100	100	100
LSDV074	Putative IMV envelope protein	100	100	99.7–100	99.7–100
LSDV075	RNA polymerase-associated protein	100	100	99.7	99.6–100
LSDV076	Late transcription factor VLTF-4	86.5–100	100	97.8–100	86.5–100
LSDV079	mRNA capping enzyme large subunit	99.9–100	100	99.9–100	99.9–100
LSDV080	Hypothetical protein	100	100	100	99.4–100
LSDV081	Putative virion protein	100	100	100	98.0–100
LSDV083	Putative NTPase	100	100	99.6–100	100
LSDV089	mRNA capping enzyme small subunit	100	100	99.7–100	99.7–100
LSDV090	Putative rifampicin resistance protein	99.8–100	100	99.7–100	99.8–100
LSDV094	Putative virion core protein	100	100	99.8–100	99.8–100
LSDV095	Virion core protein	100	100	99.8–100	100
LSDV096	RNA polymerase subunit	100	100	99.4–100	100
LSDV097	Hypothetical protein	99.7–100	100	99.4–100	99.7–100
LSDV098	Putative early transcription factor large subunit	100	100	99.7–100	100
LSDV100	Putative IMV membrane protein	100	100	99.7–100	100
LSDV102	Hypothetical protein	100	100	99.9–100	99.7–100
LSDV103	Putative virion core protein	98.4–100	100	99.5–100	98.4–99.5
LSDV104	Putative IMV membrane protein	100	100	99.5–10	100
LSDV107	Hypothetical protein	100	100	98.9–100	98.9–100
LSDV108	Putative myristylated membrane protein	100	100	98.9–100	100
LSDV109	Putative phosphorylated IMV membrane protein	100	100	99.5–100	99.5–100
LSDV110	Putative DNA helicase transcriptional elongation factor	100	100	99.4–99.6	99.6–100
LSDV111	Hypothetical protein	100	100	99.6–100	100
LSDV113	IMV membrane protein	100	100	98.3–100	100
LSDV113	Putative DNA polymerase processivity factor	100	100	99.5–100	99.8–100
LSDV114	Hypothetical protein	94.4–100	100	93.9–99.8	93.9–94.4
LSDV115	Putative intermediate transcription factor subunit	100	100	93.9–99.5	99.5–100
LSDV116	RNA polymerase subunit	100	100	99.5–100	100
LSDV122	EEV glycoprotein	100	100	99.0–100	99.0–100
LSDV123	IEV and EEV membrane glycoprotein	100	100	99.0–100	100
LSDV126	EEV glycoprotein	73.8–100	100	94.5–100	73.8–100
LSDV127	Hypothetical protein	100	100	94.5–100	99.6–100
LSDV128	CD47-like protein	99.7–100	100	98.0–99.7	99.7–100
LSDV129	Hypothetical protein	100	100	98.0–100	98.4–100
LSDV130	Hypothetical protein	100	100	96.3–100	96.3–100
LSDV131	Superoxide dismutase-like protein	93.6–100	100	96.3–100	93.6–100
LSDV132	Hypothetical protein	100	100	97.7–100	97.7–100
LSDV133	DNA ligase-like protein	100	100	97.7–99.8	99.8–100
LSDV134	Variola virus B22R-like protein	60.6–100	100	99.6–99.8	60.6–99.7
LSDV135	Putative IFN-alpha|beta binding protein	100	100	98.9	98.9–100
LSDV136	Hypothetical protein	100	100	98.9–99.3	99.3–100
LSDV137	Hypothetical protein	100	100	99.3–99.4	99.4–100
LSDV138	Ig domain OX-2-like protein	100	100	98.9–99.4	98.9–100
LSDV139	Putative ser-thr protein kinase	100	100	95.7–99.0	99.0–100
LSDV140	N1R-p28-like protein	100	100	98.3–99.0	98.3–100
LSDV141	EEV host range protein	100	100	98.3–99.6	99.1–100
LSDV142	Putative secreted virulence factor	100	100	98.5–99.1	98.5–100
LSDV143	Tyrosine protein kinase-like protein	97.1–97.1	97.1	96.5–98.5	96.5–97.1
LSDV144	Kelch-like protein	98.9–99.3	100	96.5–100	98.9–100
LSDV145	Ankyrin repeat protein	100	100	99.1–100	99.8–100
LSDV146	Phospholipase D-like protein	100	100	99.3–99.7	99.3–100
LSDV147	Ankyrin repeat protein	100	100	99.3–1,100	100
LSDV148	Ankyrin-like protein	100	100	100	99.6–100
LSDV149	Serpin-like protein	100	100	99.7–100	99.4–100
LSDV150	Hypothetical protein	100	100	99.7–100	100
LSDV151	Kelch-like protein	99.3–100	100	99.3–100	97.8–99.6
LSDV152	Ankyrin-like protein	100	100	99.2–99.6	98.4–100
LSDV153	Hypothetical protein	100	100	99.2–100	100
LSDV154	Putative ER-localized apoptosis regulator	99.6	100	100	100
LSDV155	Hypothetical protein	97.7	100	100	100

A total of 17 variable loci in the genomic sequence, resulting in variations in 7 ORFs and 2 non-coding regions, were found between LSDV/China/XJ01/2019 and the most closely related strain, China/GD01/2020. The changed ORFs represent seven proteins, hypothetical protein (LSDV001), putative myristylated protein (LSDV059), superoxide dismutase-like protein (LSDV131), tyrosine protein kinase-like protein (LSDV143), kelch-like protein (LSDV144), putative ER-localized apoptosis regulator (LSDV154), and hypothetical protein (LSDV155). In total, seven ORFs were missing in the LSDV/China/XJ01/2019 genome compared to the China/GD01/2020 genome. Of these seven missing ORFs, all (LSDV004, LSDV023, LSDV044, LSDV055, LSDV057, LSDV106 and LSDV107) had both start and stop codons in the China/GD01/2020 strain, which may be annotation issues.

The LSDV/China/XJ01/2019 strain shared 153 identical ORFs with the Vietnamese strains (20L42_Quyet-Thang/VNM/20, 20L43_Ly-Quoc/VNM/20, 20L70_Dinh-To/VNM/20 and 20L81_Bang-Thanh/VNM/20) and 3 ORFs had 97.1–99.7% sequence identities. The changed ORFs included three proteins, putative myristylated protein (LSDV059), putative viral membrane protein (LSDV073) and tyrosine protein kinase-like protein (LSDV143). The LSDV/China/XJ01/2019 strain showed no additional ORFs compared to the Vietnamese strains.

A comparison of LSDV/China/XJ01/2019 with the Russian strains (LSDV/Russia/Dagestan/2015, LSDV/Russia/Saratov/2017 and LSDV/Russia/Udmurtiya/2019), identified 75 identical ORFs and 81 ORFs sharing 60.6–99.9% sequence identities. Two ORFs (LSDV114 and LSDV131) in LSDV/Russia/Dagestan/2015 and one ORF (LSDV081) in LSDV/Russia/Udmurtiya/2019 were missing in our genomic sequence.

A comparison of LSDV/China/XJ01/2019 with the Kazakhstani strains (Kubash/KAZ/16, Neethling-RIBSP, and KZ-Kostanay-2018), revealed 53 identical ORFs and 103 ORFs sharing 75.2–99.9% sequence identities. In total, 1 ORF (LSDV023) in Kubash/KAZ/16, 10 ORFs (LSDV001, LSDV002, LSDV003, LSDV004, LSDV023, LSDV027, LSDV135, LSDV148, LSDV155, and LSDV156) in Neethling-RIBSP and 10 ORFs (LSDV001, LSDV002, LSDV003, LSDV004, LSDV114, LSDV131, LSDV153, LSDV154, LSDV155, and LSDV156) in KZ-Kostanay-2018 were missing in the genomic sequence due to lacking of the complete 5′- and 3′-terminal sequences of Neethling-RIBSP and KZ-Kostanay-2018.

### 3.5. Phylogenetic analyses

**Figure 3:**
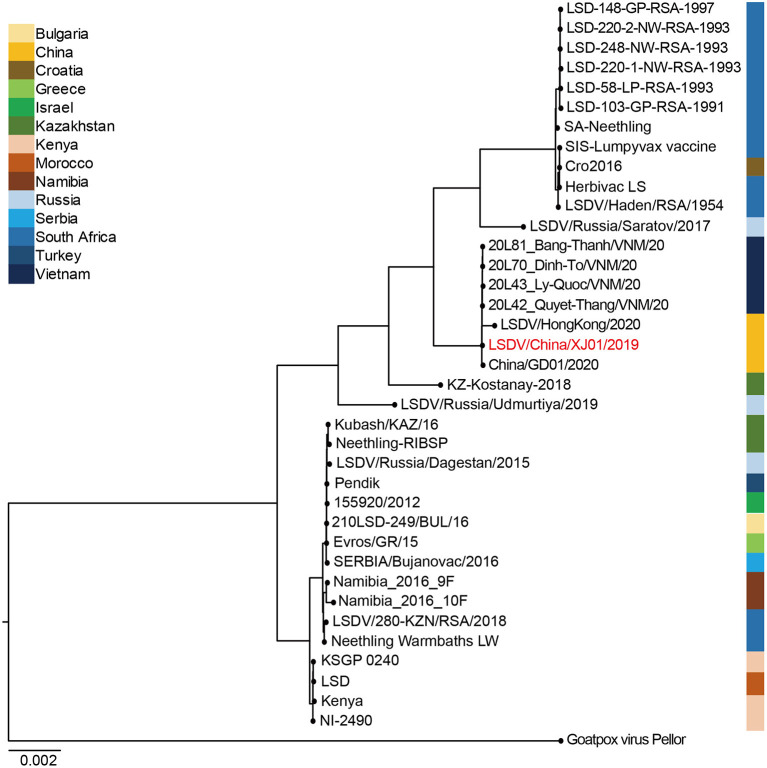
Bayesian maximum clade credibility tree of whole-genome sequences of Lumpy skin disease virus. The whole-genome MCC tree was constructed using the BEAST software package (v. 2.4.3) and then visualized by FigTree (v1.4.3). The LSDV genome sequenced in this study are labeled in red.

LSDV/China/XJ01/2019 together with China/GD01/2020, LSDV/HongKong/2020, 20L42_Quyet-Thang/VNM/20, 20L43_Ly-Quoc/VNM/20, 20L70_Dinh-To/VNM/20, and 20L81_Bang-Thanh/ VNM/20 belonged to the same clade, showing their elevated similarity and comprised a monophyletic group with short tree branches. The phylogenetic tree confirmed that Chinese and Vietnamese strains belong to the same evolutionary lineage compared to other LSDV strains (Figure 3).

## 4. Discussion

LSD is a severe systemic disease that was first reported in China 3 years ago, and it has severely impacted the cattle-breeding industry and brought significant economic losses to the affected areas (11). Before and after it, LSD outbreaks had occurred in several Eurasian countries, including Vietnam, Kazakhstan, Russia, India, and South Korea, and affected cattle, Korean water deer, and giraffe (4, 29–36), indicating a wave of highly contagious epidemic. Here we present the genomic characterization of the virulent strain of LSDV, together with its homologous viruses that were subsequently discovered in China and Vietnam. This study provides additional genomic data for LSDV evolution and is crucial for virus tracking and vaccine development.

Genomic comparisons of the LSDV/China/XJ01/2019 strain with those in and around China showed that it showed the highest level of genomic similarity with the Chinese and Vietnamese strains. Phylogenetic analysis and genomic similarity comparisons using whole genome sequences indicated that the strain LSDV/China/XJ01/2019 was most closely related to the strains China/GD01/2020, LSDV/HongKong/2020, 20L42_Quyet-Thang/VNM/20, 20L43_Ly-Quoc/VNM/20, 20L70_Dinh-To/VNM/20 and 20L81_Bang-Thanh/VNM/20, inferred that these LSDV strains might originated from a common ancestor. Genome sequencing of more LSDV strains circulating in East and Southeast Asia may help pinpoint their origins.

A total of 17 variable loci in the genomic sequence caused variations in 7 ORFs and 2 non-coding regions between LSDV/China/XJ01/2019 and the most closely related strain, China/GD01/2020. The changed ORFs encoded seven proteins, which are hypothetical protein (LSDV001), putative myristylated protein (LSDV059), superoxide dismutase-like protein (LSDV131), tyrosine protein kinase-like protein (LSDV143), kelch-like protein (LSDV144), putative ER-localized apoptosis regulator (LSDV154) and hypothetical protein (LSDV155). According to the whole-genome comparison of LSDV, the putative myristylated protein (LSDV059) and tyrosine protein kinase-like protein (LSDV143) were highly variable, indicating that the diversity of these two proteins was probably resulted from adaptive evolutionary pressure. In future, it would be instructive to reveal the association between gene mutations and viral pathogenicity or transmissibility, which would be helpful in understanding LSDV adaptive evolution.

Data on the movements of cattle into the epidemic zone during the first LSD outbreak in China and on LSDV-infected cattle in region bordering China and Kazakhstan are still lacking, indicating the emergence of LSDV in Xinjiang, northwest China may be unexpectedly complex and difficult to trace, as it is still unknown whether the outbreak was imported or localized, highlighting the need for further research. Moreover, the phylogeny of the LSDV strains in Xinjiang, northwest China, and Vietnam, which are thousands of kilometers apart, highlights how little we know about the spread of this lineage and its introduction to these regions.

In summary, we analyzed the LSDV genome from a dairy cow that died during the first LSD outbreak in China and found that the strain LSDV/China/XJ01/2019 strain was genetically close to additional LSDV strains in China and Vietnam. Molecular epidemiological investigations of LSDV in susceptible animals and vectors at national, regional, and global levels are desired to understand the evolution and transmission routes of the latest global LSD epidemic.

## Data availability statement

The data presented in the study is deposited in the Genbank, accession number OM105589.

## Ethics statement

Ethical review and approval was not required for the animal study because specimens were collected from a dead cow, but it was approved by the Animal Husbandry and Veterinary Bureau of Xinjiang Uygur Autonomous Region. Written informed consent was obtained from the owners for the participation of their animals in this study.

## Author contributions

JH conceptualized the study. Y-RW, W-GM, PW, WW, X-HS, X-YY, X-YM, and J-YW carried out the laboratory assays. Y-RW analyzed the data and drafted the paper. All authors approved the final version of the manuscript to be published.
