# Supplementary figures and images for: Retrospective genomic analysis of the first Lumpy skin disease virus outbreak in China (2019)
Source: Front Vet Sci. 2023 Jan 12;9:1073648. doi: 10.3389/fvets.2022.1073648 (PMC9879060; doi:10.3389/fvets.2022.1073648)

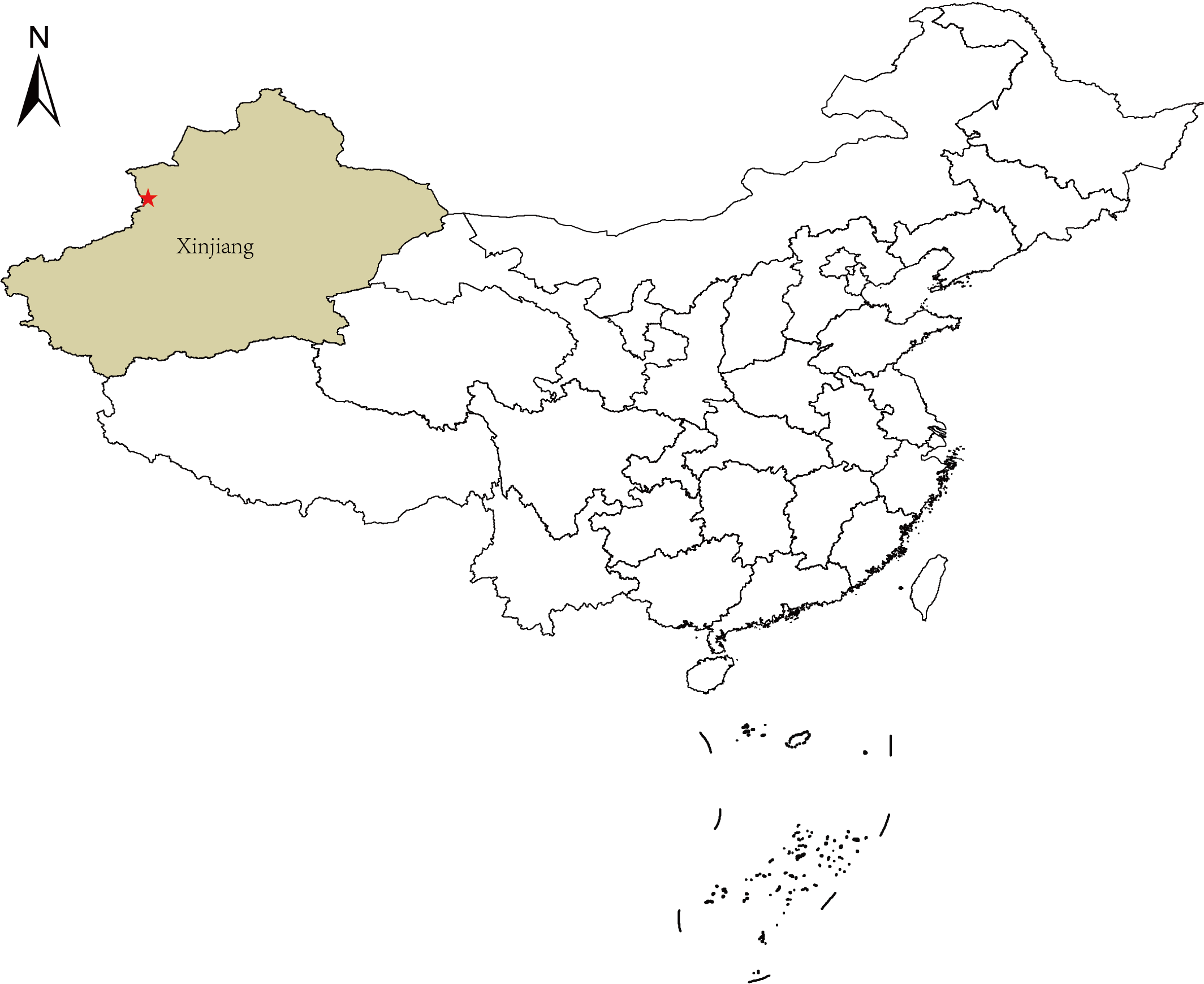

Supplement: Supplementary file 1 [file Image_1.TIF]
