# Supplementary material for: Retrospective genomic analysis of the first Lumpy skin disease virus outbreak in China (2019)
Source: Front Vet Sci. 2023 Jan 12;9:1073648. doi: 10.3389/fvets.2022.1073648 (PMC9879060; doi:10.3389/fvets.2022.1073648)
Supplement: Supplementary file 2 [file Table_1.docx]

# TABLE S1. Primers used for Lumpy Skin Disease Virus identification in this study

| **Gene** | **Primer** | **Primer sequence (5’→3’)** | **Length (bp)** | **Reference** |
| --- | --- | --- | --- | --- |
| *Ankyrin repeat protein* | Arp-F | ATGGATATTGATAATATATATAATTATATAG | 1470 | In this study |
|  | Arp-R | CTATTTTTTATTATTTTTCAAAAACA |  |  |
| *Interleukin-1 receptor-like protein* | IRP-F | TTATATTTCATTTTTGTAAAGGGT | 1026 | In this study |
|  | IRP-R | ATGGAAAAAGTAACGACATTTA |  |  |
| *Putative alpha amanitin-sensitive protein* | Aasp-F | TTATGGAAATCTATCACAGATA | 693 | In this study |
|  | Aasp-R | ATGTATACAAAAAATAAATTTAAAATCT |  |  |
| *Putative late transcription factor* | LTF-F | ATGTCGTGGTCTATAAATTTG | 672 | In this study |
|  | LTF-R | TTACTTTTTAGTGTTTTTAACTT |  |  |
| *RNA polymerase 30 kDa subunit* | RPO30-F | ATGGCAAAAATGGAAAATATTAC | 1134 | In this study |
|  | RPO30-R | TTAATTGTTTTTTGCTATAGTCA |  |  |
| *G-protein-coupled chemokine receptor* | GPRC-F | TTAAGTAAAGCATAACTCCAACAAAAATG | 1159 | [13] |
|  | GPRC -R | TTTTTTTATTTTTTATCCAATGCTAATACT |  |  |
