# Supplementary material for: Retrospective genomic analysis of the first Lumpy skin disease virus outbreak in China (2019)
Source: Front Vet Sci. 2023 Jan 12;9:1073648. doi: 10.3389/fvets.2022.1073648 (PMC9879060; doi:10.3389/fvets.2022.1073648)
Supplement: Supplementary file 3 [file Table_2.docx]

# TABLE S2. Sequences of Lumpy Skin Disease Virus strains used for construction of the Maximum Likelihood tree

| **Strain designation** | **Virus species** | **Accession nos.** | **Year** | **Country** |
| --- | --- | --- | --- | --- |
| 210LSD-249/BUL/16 | LSDV^*^ | MT643825.1 | 2016 | Bulgaria |
| China/XJ/2019 | LSDV | MN508357.1 | 2019 | China |
| China/XJ/flies/2019 | LSDV | MN864147.1 | 2019 | China |
| Cro2016 | LSDV | MG972412.1 | 2016 | Croatia |
| Evros/GR/15 | LSDV | KY829023.3 | 2015 | Greece |
| 155920/2012 | LSDV | KX894508.1 | 2012 | Israel |
| Kubash/KAZ/16 | LSDV | MN642592.1 | 2016 | Kazakhstan |
| Neethling-RIBSP | LSDV | MT130502.2 | 2018 | Kazakhstan |
| KZ-Kostanay-2018 | LSDV | MT992618.1 | 2018 | Kazakhstan |
| Kazakhstan/2016 | LSDV | MK765544.1 | 2016 | Kazakhstan |
| NI-2490 isolate Neethling 2490 | LSDV | AF325528.1 | 1958 | Kenya |
| KSGP 0240 | LSDV | KX683219.1 | 1974 | Kenya |
| Kenya | LSDV | MN072619.1 | 1958 | Kenya |
| NI-2490 | LSDV | NC_003027.1 | 1958 | Kenya |
| LSD | LSDV | MW631933.1 | 2017 | Morocco |
| Namibia_2016_9F | LSDV | MT007950.1 | 2016 | Namibia |
| Namibia_2016_10F | LSDV | MT007951.1 | 2016 | Namibia |
| LSDV8 | LSDV | FJ869368.1 | — | Nigeria |
| LSDV/Russia/Saratov/2017 | LSDV | MH646674.1 | 2017 | Russia |
| LSDV/Russia/Dagestan/2015 | LSDV | MH893760.2 | 215 | Russia |
| LSDV/Russia/Udmurtiya/2019 | LSDV | MT134042.1 | 2019 | Russia |
| Omsk/2018 | LSDV | MK765545.1 | 2018 | Russia |
| Saratov/Nesterovo-30840/2019/bl_3 | LSDV | MT129673.1 | 2019 | Russia |
| Omsk/2019 | LSDV | MT395338.1 | 2019 | Russia |
| Saratov/2019 | LSDV | MT395339.1 | 2019 | Russia |
| SERBIA/Bujanovac/2016 | LSDV | KY702007.1 | 2016 | Serbia |
| NW-LW isolate Neethling Warmbaths LW | LSDV | AF409137.1 | 1999 | South Africa |
| Neethling vaccine LW 1959 | LSDV | AF409138.1 | 1959 | South Africa |
| SIS-Lumpyvax vaccine | LSDV | KX764643.1 | 1999 | South Africa |
| Neethling-Herbivac vaccine | LSDV | KX764644.1 | — | South Africa |
| Neethling-LSD vaccine-OBP | LSDV | KX764645.1 | — | South Africa |
| Herbivac LS | LSDV | MK441838.1 | 2011 | South Africa |
| LSD-58-LP-RSA-1993 | LSDV | MN636838.1 | 1993 | South Africa |
| LSD-103-GP-RSA-1991 | LSDV | MN636839.1 | 1991 | South Africa |
| LSD-248-NW-RSA-1993 | LSDV | MN636840.1 | 1993 | South Africa |
| LSD-220-1-NW-RSA-1993 | LSDV | MN636841.1 | 1993 | South Africa |
| LSD-220-2-NW-RSA-1993 | LSDV | MN636842.1 | 1993 | South Africa |
| LSD-148-GP-RSA-1997 | LSDV | MN636843.1 | 1997 | South Africa |
| SA-Neethling | LSDV | MW435866.1 | 1959 | South Africa |
| LSDV/Haden/RSA/1954 | LSDV | MW656252.1 | 1954 | South Africa |
| LSDV/280-KZN/RSA/2018 | LSDV | MW656253.1 | 2018 | South Africa |
| Pendik | LSDV | MN995838.1 | 2014 | Turkey |
| TU-V02127 | Sheeppox virus | AY077832.1 | 1970 | Turkey |
| Gorgan | Goatpox virus | KX576657.1 | 1965 | Iran |
| G20-LKV | Goatpox virus | AY077836.1 | 2000 | Kazakhstan |
| A | Sheeppox virus | AY077833.1 | 1987 | Kazakhstan |
| NISKHI | Sheeppox virus | AY077834.1 | 1994 | Kazakhstan |

^*^ Abbreviation: LSDV, Lumpy skin disease virus.
