# Supplementary material for: Retrospective genomic analysis of the first Lumpy skin disease virus outbreak in China (2019)
Source: Front Vet Sci. 2023 Jan 12;9:1073648. doi: 10.3389/fvets.2022.1073648 (PMC9879060; doi:10.3389/fvets.2022.1073648)
Supplement: Supplementary file 4 [file Table_3.docx]

# TABLE S3. Genome sequences of Lumpy skin disease virus strains used in this study

| **Strain designation** | **Year** | **Country** | **Host species** | **Accession** **no.** | **Reference** |
| --- | --- | --- | --- | --- | --- |
| 210LSD-249/BUL/16 | 2016 | Bulgaria | *Bos taurus* | MT643825.1 | Unpublished |
| China/GD01/2020 | 2020 | China | *Bos taurus* | MW355944.1 | [25] |
| LSDV/China/XJ01/2019 | 2019 | China | *Bos taurus* |  | In this study |
| LSDV/HongKong/2020 | 2020 | China | *Bos taurus* | MW732649.1 | [20] |
| Cro2016 | 2016 | Croatia | *Bos taurus* | MG972412.1 | Unpublished |
| Evros/GR/15 | 2015 | Greece | *Bos taurus* | KY829023.3 | [42] |
| 155920/2012 | 2012 | Israel | *Bos taurus* | KX894508.1 | Unpublished |
| Kubash/KAZ/16 | 2016 | Kazakhstan | *Bos taurus* | MN642592.1 | Unpublished |
| Neethling-RIBSP | 2018 | Kazakhstan | *Bos taurus* | MT130502.2 | Unpublished |
| KZ-Kostanay-2018 | 2018 | Kazakhstan | *Bos taurus* | MT992618.1 | Unpublished |
| Kenya | 1958 | Kenya | *Bos taurus* | MN072619.1 | [43] |
| KSGP 0240 | 1974 | Kenya | *Ovis aries* | KX683219.1 | Unpublished |
| NI-2490 | 1958 | Kenya | *Bos taurus* | NC_003027.1 | [22] |
| LSD | 2017 | Morocco | *Bos taurus* | MW631933.1 | Unpublished |
| Namibia_2016_10F | 2016 | Namibia | *Bos taurus* | MT007951.1 | Unpublished |
| Namibia_2016_9F | 2016 | Namibia | *Bos taurus* | MT007950.1 | Unpublished |
| LSDV/Russia/Dagestan/2015 | 2015 | Russia | *Bos taurus* | MH893760.2 | [3] |
| LSDV/Russia/Saratov/2017 | 2017 | Russia | *Bos taurus* | MH646674.1 | Unpublished |
| LSDV/Russia/Udmurtiya/2019 | 2019 | Russia | *Bos taurus* | MT134042.1 | Unpublished |
| SERBIA/Bujanovac/2016 | 2016 | Serbia | *Bos taurus* | KY702007.1 | [44] |
| Herbivac LS | 2011 | South Africa | *Bos taurus* | MK441838.1 | Unpublished |
| LSD-103-GP-RSA-1991 | 1991 | South Africa | *Bos taurus* | MN636839.1 | [45] |
| LSD-148-GP-RSA-1997 | 1997 | South Africa | *Bos taurus* | MN636843.1 | [45] |
| LSD-220-1-NW-RSA-1993 | 1993 | South Africa | *Bos taurus* | MN636841.1 | [45] |
| LSD-220-2-NW-RSA-1993 | 1993 | South Africa | *Bos taurus* | MN636842.1 | [45] |
| LSD-248-NW-RSA-1993 | 1993 | South Africa | *Bos taurus* | MN636840.1 | [45] |
| LSD-58-LP-RSA-1993 | 1993 | South Africa | *Bos taurus* | MN636838.1 | [45] |
| Neethling Warmbaths LW | 2001 | South Africa | *Bos taurus* | AF409137.1 | [46] |
| SA-Neethling | 1959 | South Africa | *Bos taurus* | MW435866.1 | Unpublished |
| LSDV/Haden/RSA/1954 | 1954 | South Africa | *Bos taurus* | MW656252.1 | [45] |
| LSDV/280-KZN/RSA/2018 | 2018 | South Africa | *Bos taurus* | MW656253.1 | [45] |
| SIS-Lumpyvax vaccine | 1999 | South Africa | *Bos taurus* | KX764643.1 | Unpublished |
| Pendik | 2014 | Turkey | *Bos taurus* | MN995838.1 | Unpublished |
| 20L42_Quyet-Thang/VNM/20 | 2020 | Vietnam | *Bos taurus* | MZ577073.1 | Unpublished |
| 20L43_Ly-Quoc/VNM/20 | 2020 | Vietnam | *Bos taurus* | MZ577074.1 | Unpublished |
| 20L70_Dinh-To/VNM/20 | 2020 | Vietnam | *Bos taurus* | MZ577075.1 | Unpublished |
| 20L81_Bang-Thanh/VNM/20 | 2020 | Vietnam | *Bos taurus* | MZ577076.1 | Unpublished |
